# Supplementary material for: Iron–Gold Nanoflowers: A Promising Tool for Multimodal Imaging and Hyperthermia Therapy
Source: Pharmaceutics. 2022 Mar 14;14(3):636. doi: 10.3390/pharmaceutics14030636 (PMC8955043; doi:10.3390/pharmaceutics14030636)
Supplement: Supplementary file 1 [file pharmaceutics-14-00636-s001.zip › pharmaceutics-1603448-supplementary.pdf]

pharmaceutics

MDPI

Supplementary Materials: Iron–Gold Nanoflowers: A Promising Tool for Multimodal Imaging and Hyperthermia Therapy

Evangelia Christou, John R. Pearson, Ana M. Beltrán, Yilian Fernández-Afonso, Lucía Gutiérrez, Jesús M. de la Fuente, Francisco Gámez, María L. García-Martín and Carlos Caro

1. Methods.

A) Synthesis of Iron Oleate

A mixture of 10.8 g of iron chloride (40 mmol) and 36.5 g of sodium oleate (120 mmol) were dissolved in 80 mL of ethanol, 60 mL of distilled water and 140 mL of hexane. The resulting solution was heated till 60 °C and left for 4 h allowing a reflux of hexane in an inert atmosphere. The reaction was then cooled down to room temperature and two phases could be distinguished: a lower aqueous phase and an upper organic phase containing the iron oleate. The organic phase was washed 3 times with distilled water and the hexane evaporated in a rotavapor.

B) Synthesis of PEGylated Ligand.

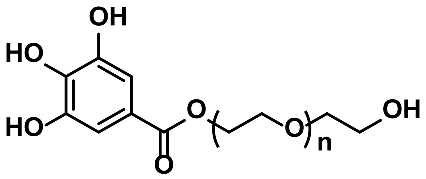

Briefly, to a solution of polyethylene glycol (Mw: 3000 g/mol, 1 mmol, 3.0 g), gallic acid (Mw: 170 g/mol, 1 mmol, 170 mg) and 4-(dimethylamino) pyridine (Mw: 122 g/mol, 200 µmol, 24 mg) in 100 mL of tetrahydrofuran and 10 mL of dichloromethane, in a round-bottom flask under nitrogen atmosphere, a solution of dicyclohexyl carbodiimide (Mw: 206 g/mol, 5 mmol, 1 g) in tetrahydrofuran was added dropwise. The mixture was stirred overnight at room temperature. The reaction mixture was filtered through filter paper and the solvents rota-evaporated.

C) Ligand Exchange

Briefly, 50 µL of triethylamine was added to a glass vial containing a 1.0 mL solution of NPs (10 g/L of Fe) and 1.0 mL of 0.1 M gallol-PEGn-OH derived in $CHCl_3$ The mixture was ultrasonicated for 1 h and heat to 50 °C for 4 h. It was then diluted with 5 ml of toluene, 5 mL of milli-Q water and 10 mL of acetone. This mixture was shaken and the nanoparticles were transferred into the aqueous phase. The aqueous phase was collected in a round-bottomed flask and the residual organic solvents rota-evaporated. The gallol derived NPs were then purified in centrifuge filters with a molecular weight cut-off of 100 kDa at 450 rcf. In each centrifugation, the functionalized NPs were re-suspended with milli-Q water. The purification step was repeated several times until the filtered solution was clear. After purification, the gallol derived NPs were resuspended in PBS buffer. Finally, to ensure highly stable mono-dispersed NPs, this solution was centrifuged at 150 rcf for 5 min and placed onto a permanent magnet (0.6 T) for 5 min.

D) Characterization Methodology

Transmission Electron Microscopy (TEM)

TEM images were obtained on a FEI Tecnai G2 Twin microscope operated at an accelerating voltage of 100 kV. TEM samples were prepared by placing a solution of the

corresponding nanoparticles at ~1 g/ L of (Fe+Au) dropwise onto a carbon-coated copper grid and letting the solvent evaporate. Size measurements were calculated as the average of 100 individual nanoparticles in each case.

Scanning-Transmission Electron Microscopy (STEM)

STEM images were obtained on a FEI (Thermofischer, USA) TALOS F200 with an accelerating voltage of 200 kV and equipped with a super-x energy dispersive x-ray spectrometry (EDX) system, which includes two silicon drift detectors. Compositional analyses of the samples were performed by combining high-angle annular dark-field imaging (HAADF). Samples were prepared by placing 100 μL of the corresponding NPs solution at ~1 $g{\cdot}L^{-1}$ of (Fe+Au) dropwise onto a carbon-coated copper grid.

X-ray Powder Diffraction (XRD)

The patterns were obtained using a Panalytical EMPYREAN diffractometer (Cu Kα) with a PIXcel detector with a 2θ step.

Inductively Coupled Plasma High-Resolution Mass Spectroscopy (ICP-HRMS)

Fe, Co, and Au concentrations were determined on a NexION ICP-HRMS (Perkin-Elmer, Waltham, MA, USA). Briefly, 2.5 mL of aqua regia were added to 25 μL of a solution of NPs in a volumetric flask. The mixture was left overnight. Then, milli-Q water was added to a total volume of 25 mL.

UV-Vis Spectroscopy

The UV-Vis spectra were recorded in a UV-VIS-NIR Spectrophotometer (Jasco V670) with a quartz tray with a light path of 1 cm. Nanoparticles were used at a concentration of ≈ 0.04 mg/mL

Fourier Transform Infra-Red Spectroscopy (FTIR)

FTIR spectra were recorded with a FTIR-4100 Jasco using a single reflection ATR accessory (MIRacle ATR, PIKE Technologies) coupled to a liquid nitrogen-cooled mercury cadmium telluride (MCT) detector. All spectra were recorded in the 4000 to 800 cm^{-1} range at 2 cm^{-1} resolution and accumulating 200 scans. All samples were recorded as solid products.

Dynamic Light Scatering (DLS)

The size distribution and zeta potential measurements of the gallol derived MNPs were performed on a Zetasizer Nano ZS90 (Malvern, USA). The nanoparticles were dispersed in NaCl at a concentration of 100 mg/L of Fe+Au. The measurements were done on a cell type: ZEN0118-low volume disposable sizing cuvette, setting 2.420 as refractive index with 173° Backscatter (NIBS default) as angle of detection. The measurement duration was set as automatic and three as the number of measurements. A general purpose analysis model was chosen (normal resolution).

Magnetic Characterization

Field-dependent magnetization measurements were performed on dried powder using a Quantum Design (USA) MPMS-XL Superconducting Quantum Interference Device (SQUID) magnetometer at 300 K with a maximum field of 0.2 T.

Relaxivity Measurements

^{1}H NMR relaxation time, T_1, was measured at low (1.44 T) magnetic field, using magnetic NPs in PBS with Fe concentrations ranging from 0.125 to 2 mM in physiological conditions at 37 °C. T_1 measurements at low magnetic field were performed on a Bruker Minispec system (Bruker BioSpin, Rheinstetten, Germany). T_1 was determined using an inversion-recovery sequence.

T_2 was measured at low and high (9.4 T) magnetic fields, with same concentration as described previously. T_2 measurements at low magnetic field were performed using the Carl-Purcell-Meiboom-Gill (CPMG) sequence on a Bruker Minispec system. At high field, T_2values was measured on a Bruker Biospec MRI system (Bruker Biospec, Bruker BioSpin, Ettlingen, Germany) equipped with 400 mT m^{-1} field gradients and a 40 mm quadrature bird-cage resonator at 298 K. T_2 values were measured using a 64-echo Carl-Purcell-Meiboom-Gill (CPMG) imaging sequence (TE values from 7.5 ms to 640 ms). The relaxivity, r_2, at both magnetic fields was calculated from the slope of the linear fit of the relaxation rate ($1/T_2$) versus the concentration.

Regions of interest (ROIs) were drawn on the first image of the image sequence and the intensity values extracted and fit to the following equations:

$$M_Z(t) = M_0(1 - e^{-TR/T_1})$$

$$M_{XY}(t) = M_0 e^{-TE/T_2}$$

Where M_z and M_{xy} are the signal intensities at time TR or TE, and M_0 is the signal intensity at equilibrium.

In Vitro X-ray Attenuation Measurement

CT images were acquired on a Bruker Albira small animal CT system (Bruker Biospec, Bruker BioSpin, Ettlingen, Germany). The X-ray Focal Spot Size (Nominal) used was 35 μm and the Energy used was 45 kVp, working at 400 μA. X-ray attenuation coefficients in Hounsfield units (HU) were plotted against gold concentrations to calculate the slope.

Photothermal Conversion

The photothermal conversion was studied by measuring the temperature variation over time of the solution (1 $g \cdot L^{-1}$) under IR irradiation. The NPs suspension was placed into a 1 cm optical path quartz cuvette and irradiated for 10 min using a laser of 1064 nm (Laser Quantum, mpc6000/Ventus 1064) while recording the suspension temperature with a fiber optic sensor (TPT-62, FISO). After that time, the laser was turned off, and the heat release was recorded for 30 min. The laser power was set to 1.22 W. The optical Specific Absorption Rate (oSAR) was calculated from the experimental photothermal conversion data (in $W \cdot g^{-1}$). oSAR is defined here as the power dissipation per unit mass of NP (m_{NP}):

$$oSAR = \frac{CV}{m_{NP}} \times \frac{dT}{dt}$$

where dT/dt is the temperature increase as a function of time and it was calculated at a time t = 10 min. C is the specific heat capacity of the sample, and V is the total volume.

Magnetic Specific Absorption Rate (mSAR) Measurement

SAR measurements of the nanoparticles were performed by using DM5 equipment (Nanoscale Biomagnetics). Applied magnetic field frequency was set at 145.2 kHz and the magnetic field amplitude was 580 Gauss or 46.16 kA/m. Samples were placed in a magnetic induction coil cooled by water.

E) Cytotoxicity Evaluation

Cell morphology studies and "live-dead" assay. The HFF-1 cells were plated at a density of 1 × 10^4 cells/well in a 96-well plate at 37 °C in 5% CO_2 atmosphere (100 μL per well, number of repetitions = 5). After 24 h of culture, the medium in the wells was replaced by fresh media containing the magnetic NPs in varying concentrations from 0.1 to 50 μg/mL (Fe+Au). After 24 h, ethanol 20% was added to the positive control wells. All the wells were stained for15 min with: 1) Hoechst 33342 (Merck; 2.5 μg/mL final concentration) to

label cells nuclei; and 2) Propidium iodide (PI) (Merck; 2μg/mL final concentration) to label dead cells. Cell morphology images were acquired with a Perkin Elmer Operetta High Content Imaging System with a 10× LWD air objective lens. Five well replicates for each condition were analyzed with 5 image fields captured per well. For each field, fluorescence images for Hoechst 33343 and PI, plus a brightfield image, were captured. Cell mortality percentages were calculated automatically by Operetta Harmony software, whereby all nuclei (dead and alive) were identified from the Hoechst 33343 staining and the percentage of dead cells then determined by the number of nuclei presenting high levels of PI staining. Calcein-AM (1:1000 in PBS) has been used to evaluate the intracellular esterase activity for 100 μg/mL (Fe+Au).

MTT Assay

Briefly, HFF-1 cells were plated at a density of 1 × 10^4 cells/well in a 96-well plate at 37 °C in 5 % CO_2 atmosphere (200 μL per well, number of replicas= 5). After 24 h of culture, well culture media was replaced with fresh medium containing magnetic nanoparticles in concentrations varying from 0.1 μg/mL to 100 μg/mL. After 24 h, the supernatant of each well was replaced by 200 μL of fresh medium with 3-[4,5-dimethylthiazol-2-yl]-2,5-diphenyl tetrazolium bromide (MTT) (0.5 mg·mL^{-1}). After 2 h of incubation at 37 °C and 5 % CO_2, the medium was removed, the formazan crystals were solubilized with 200 μL of DMSO, and the solution was vigorously mixed to dissolve the reacted dye. The absorbance of each well $[Abs]_{well}$ was read on a microplate reader (Dynatech MR7000 instruments) at 550 nm. The relative cell viability (%) and its error related to control wells containing cell culture medium without nanoparticles were calculated by the equations:

$$RCV(\%) = \left(\frac{[Abs]_{test} - [Abs]_{Pos.Ctrl.}}{[Abs]_{Neg.Ctrl.} - [Abs]_{Pos.Ctrl.}}\right) \times 100$$

$$Error(\%) = RCV_{test} \times \sqrt{\left(\frac{\sigma_{test}}{[Abs]_{test}}\right)^2 + \left(\frac{\sigma_{Ctrl}}{[Abs]_{Ctrl.}}\right)^2}$$

where σ is the standard deviation. Triton X-100 was added to the positive control wells.

2. Results.

A) Characterization

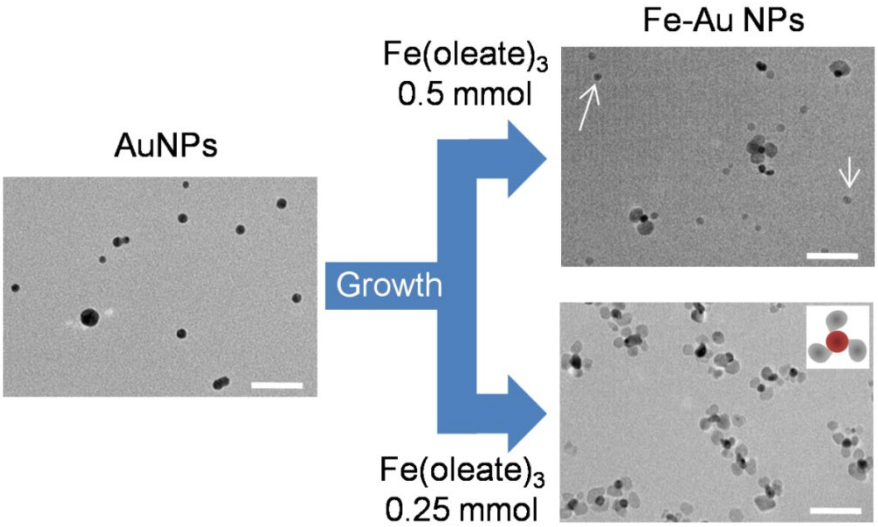

Figure S1. Representative TEM images of Au seeds and different growth processes with different amounts of iron precursor. Scale bars correspond to 50 nm.

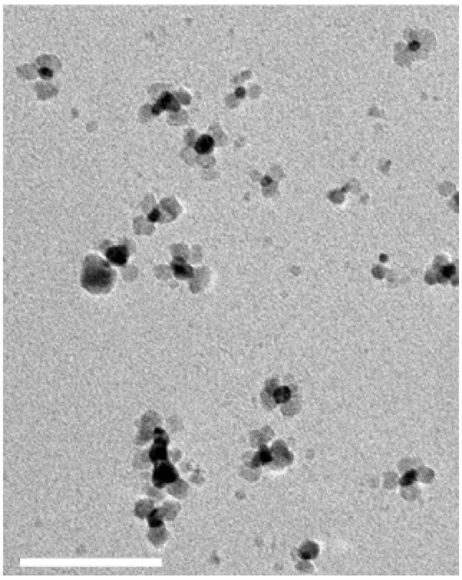

Figure S2. Representative TEM images of Au@Fe-PEG NP. Scale bars correspond to 100 nm.

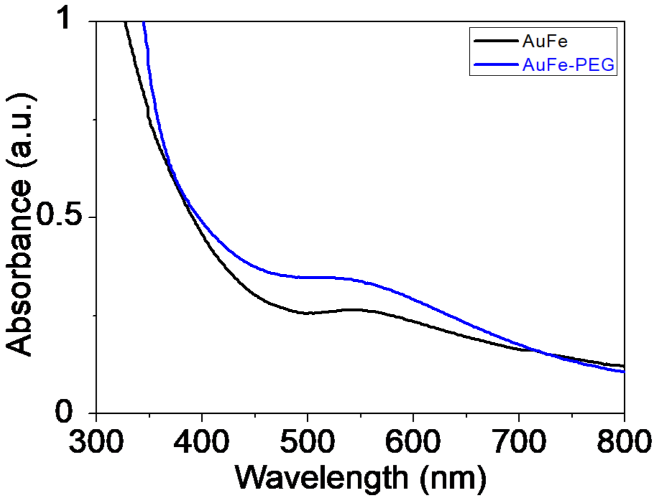

Figure S3. UV-Vis spectra of oleic acid capped Fe@Au NPs (black) and PEGylated Fe@Au NPs (blue).

B) In Vitro Transversal Relaxivity.

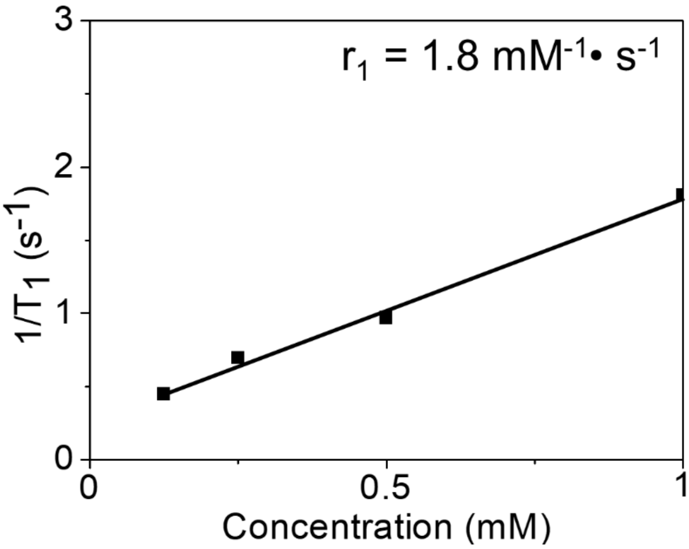

Figure S4. Plot of $1/T_1$ over Fe concentration of PEGylated Fe@Au NPs calculated at 1.44 T.

C) In Vitro Cytotoxicity Evaluation

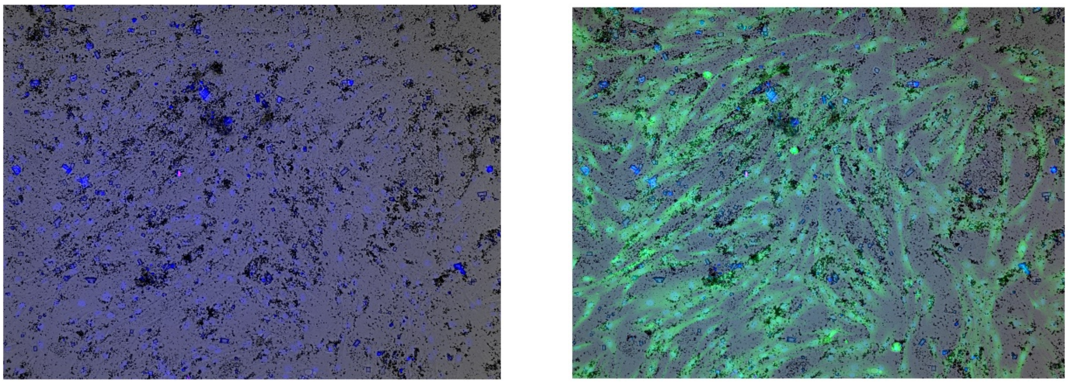

Figure S5. Representative optical microscopy images of HFF-1 cells incubated with Au@Fe-PEG NPs (100 μg/mL Fe+Au). Right image shows a merge of brightfield (grey), Hoechst 33342 (blue) and PI (red), while left image shows a merge of brightfield (grey), Hoechst 33342 (blue), PI (red) and Calcein-AM (green).
